# Exploring cannabinoid modulation on autophagy mechanisms in Alzheimer’s disease: a review

**DOI:** 10.3389/fphar.2025.1748368

**Published:** 2026-01-12

**Authors:** C. Ntsapi, M. Weyers, R. Chinheya, T. Jim, M. Matsabisa

**Affiliations:** 1 Department of Basic Medical Sciences, School of Biomedical Sciences, Faculty of Health Sciences, University of the Free State, Bloemfontein, South Africa; 2 Department of Pharmacology, School of Clinical Medicine, Faculty of Health Sciences, University of the Free State, Bloemfontein, South Africa; 3 University of the Free State – Technology Innovation Agency Pharmacology Platform (AMITD), Bloemfontein, South Africa

**Keywords:** Alzheimer’s disease, amyloid precursor protein, amyloid-β (Aβ), autophagy, cannabinoid-based therapeutics, Cannabis sativa

## Abstract

Alzheimer’s disease (AD) is a neurodegenerative disorder characterized by the accumulation of toxic protein aggregates in the brain, leading to brain cell death and cognitive impairment. Central to AD pathogenesis is the autophagy pathway, a crucial cellular self-digestion process. Cannabinoids, the fundamental phytochemical compounds derived from the *Cannabis sativa* plant, have been demonstrated to exhibit neuroprotective qualities when used as a treatment at microdoses. However, the impact of multi-cannabinoid treatments on autophagy induction and subsequent cell survival in AD *in vitro* models remains uncertain. This review seeks to explore the potential of a multi-cannabinoid treatment strategy in enhancing neuronal cell survival through autophagy activation within an AD *in vitro* model. The proposed approach involves a combination of cannabinoids in their potential to upregulate autophagy mechanisms, potentially supporting neuronal cell resilience. By unravelling the mechanistic link between autophagy, cannabinoid treatment, and neuronal viability, this review aims to elucidate how cannabinoids influence neuronal function and survival at a cellular and molecular level. By offering insights into the exploitation of the endocannabinoid system, this review contributes to the development of novel cannabinoid-based treatment avenues for AD. This pursuit aligns with the broader objective of addressing the debilitating effects of AD on the quality of life for those affected.

## 1 Introduction

Neurodegeneration involves the progressive decline of neurons and is a hallmark of disorders such as Alzheimer’s disease (AD), Parkinson’s disease (PD), and Amyotrophic Lateral Sclerosis (Mistretta et al., 2023; Agnello and Ciaccio, 2022). AD, the most prevalent neurodegenerative disorder (Świetlik et al., 2022), is characterized by the accumulation of amyloid-β (Aβ) plaques and hyperphosphorylated tau, leading to neuronal dysfunction, inflammation, and oxidative stress in the central nervous system (Halawani et al., 2010; Rohn and Head, 2009). AD is broadly classified based on its pathophysiology as either sporadic (late-onset) or familial (early-onset). Sporadic AD, accounting for approximately 95% of cases, is a complex disorder influenced by multiple factors that remain incompletely understood. These include genetic factors, such as the Apolipoprotein E allele, as well as environmental and lifestyle influences, and the interplay of epigenetic and genetic processes (Tailor et al., 2019; Ibanez et al., 2021). Familial AD, which affects fewer than 5% of patients, results from mutations in specific genes that alter the amyloid precursor protein (APP) or presenilin-1 and -2 (PS1/PS2) (Xiao et al., 2021; Lanoiselée et al., 2017). Both sporadic and familial forms of AD share these pathological features, though the underlying causes differ (Tailor et al., 2019; Ibanez et al., 2021; Lanoiselée et al., 2017; d‘Errico and Meyer-Luehmann, 2020). The amyloid cascade hypothesis suggests that impaired clearance of Aβ triggers downstream tau pathology and neuronal toxicity, as demonstrated in cellular and animal models (Hardy and Higgins, 1992; Alawode et al., 2022). Despite strong preclinical evidence, translating these findings into effective clinical therapies has been challenging, often due to safety concerns and limitations of conventional drug approaches.

Despite the strong preclinical evidence linking Aβ accumulation and tau pathology to AD progression, translating this robust preclinical and biomarker science into clinical benefit has proven challenging. Logistical difficulties, safety concerns, and limited tolerable dosages have hindered the success of secretase-inhibiting therapies, and several clinical trials have been prematurely halted due to adverse effects or other pharmaceutical limitations (Haass and Selkoe, 2022; Ricciarelli and Fedele, 2017). These challenges underscore the need for alternative therapeutic strategies, such as cannabinoids, which have shown potential in modulating neuroinflammation, oxidative stress, and Aβ- and tau-mediated neuronal toxicity, positioning them as promising multi-target agents in AD management (d‘Errico and Meyer-Luehmann, 2020; DeTure and Dickson, 2019; Halawani et al., 2010; Rohn and Head, 2009; Yankner et al., 1989).

## 2 APP processing and Aβ peptide formation

APP is a widely spread transmembrane protein (Morley et al., 2019) involved in multiple physiological processes, including wound healing, calcium regulation in neurons, transport of molecules across cell membranes, and the formation and maintenance of synapses, which are critical for neuronal communication (Morley et al., 2019; O’Brien and Wong, 2011; Bergström et al., 2016). Importantly, abnormal accumulation of APP’s proteolytic product, the Aβ peptide, is a defining feature of AD (O’Brien and Wong, 2011; Bergström et al., 2016). Aβ peptides are produced through the sequential cleavage of APP by β-secretase (BACE) and γ-secretase enzymes. This processing produces N-terminal fragments, such as soluble amyloid precursor protein-alpha (sAPPα) and soluble amyloid precursor protein-beta (sAPPβ), Aβ peptides, and C-terminal fragments (O’Brien and Wong, 2011).

**FIGURE 1:**
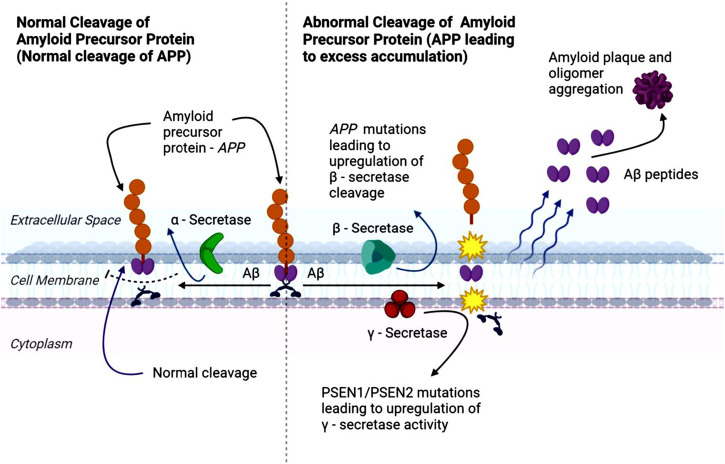
Schematic representation of the amyloid precursor protein (APP) processing cascade. The diagram illustrates the normal (left) and abnormal (right) pathways of APP cleavage. In the normal, non-amyloidogenic pathway, α-secretase cleaves APP to generate soluble fragments and prevents the formation of amyloid-β (Aβ) peptides. In Alzheimer’s disease (AD), or in the presence of pathogenic mutations in APP or the presenilin genes PSEN1 and PSEN2, APP is preferentially processed by β-secretase and γ-secretase, leading to the production of Aβ peptides that accumulate extracellularly. These peptides oligomerize and aggregate to form amyloid plaques, a neuropathological hallmark of AD.

Aβ peptides are hydrophobic and prone to aggregate, forming oligomers and protofibrils that eventually deposit as Aβ plaque in the brain (Morley et al., 2019). APP can undergo either non-amyloidogenic or amyloidogenic processing, with the latter favored in AD (Fong et al., 2018; Zhao et al., 2020). In the amyloidogenic pathway (Figure 1), β-secretase mediates the initial cleavage of APP, producing sAPPβ and a carboxy-terminal fragment β (CTFβ or C99) (Roberds et al., 2001; Hampel et al., 2021). C99 fragments are subsequently cleaved by γ-secretase, generating Aβ and carboxy-terminal fragment γ (CTFγ) or APP intracellular domain (AICD). Excessive APP cleavage drives the accumulation of Aβ fragments, which aggregate into oligomers and fibrils, culminating in plaque formation, and contributing to the synaptic dysfunction, inflammation, and neurotoxicity characteristic of AD (Roberds et al., 2001; Hampel et al., 2021; Takahashi et al., 2017).

## 3 Approved drugs for AD treatment and their mechanism of action

**FIGURE 2:**
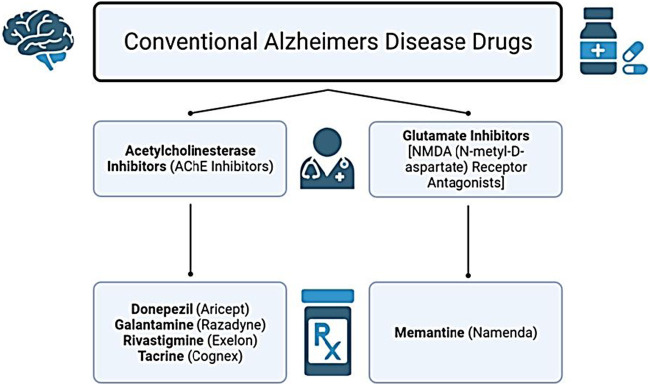
Conventional symptomatic drug classes used in Alzheimer’s disease (AD) treatment. Acetylcholinesterase inhibitors (AChEIs) - donepezil, galantamine, rivastigmine, and tacrine - enhance cholinergic signalling by increasing acetylcholine availability, providing temporary cognitive and functional benefits in mild to moderate AD. Glutamatergic modulation is achieved with the N-methyl-D-aspartate (NMDA) receptor antagonist memantine, which reduces excitotoxicity and is indicated for moderate to severe AD. These drugs offer symptomatic relief but do not modify underlying disease progression.

The growing global prevalence of AD underscores the urgent need for effective therapeutic strategies. Although scientific and medical advances have improved understanding of AD, no treatment currently exists that can halt or reverse disease progression. The disorder remains non-preventable, incurable, and challenging to diagnose with high accuracy (d‘Errico and Meyer-Luehmann, 2020; Hardy and Higgins, 1992). To date, two major classes of medications are approved for AD: acetylcholinesterase inhibitors and N-methyl-D-aspartate (NMDA) receptor antagonists (Alawode et al., 2022; Haass and Selkoe, 2022). In the United States, the Food and Drug Administration (FDA) has approved drugs that either modestly slow clinical decline or offer temporary symptomatic relief. These include the acetylcholinesterase inhibitors - Rivastigmine, Donepezil, Galantamine, and Tacrine - alongside Memantine (MEM), the primary NMDA receptor antagonist (glutamate inhibitor) (Reisberg et al., 2003). These conventional drug classes are summarized in Figure 2.

### 3.1 Acetylcholinesterase in AD treatment

Neurodegeneration in AD is driven in part by the accumulation of Aβ aggregates and hyperphosphorylated tau, leading to progressive neuronal loss and deterioration of cognitive, language, and memory functions (Sharma, 2019). Acetylcholine (ACh), a key neurotransmitter within cholinergic neurons of the central and autonomic nervous systems, plays an essential role in learning, memory, and synaptic plasticity (Radu et al., 2017; Picciotto et al., 2012). These systems include the well-known neuromuscular junctions, each exhibiting distinct nicotinic and muscarinic receptors (Picciotto et al., 2012). In the brain, all five subtypes of muscarinic receptors (M1–M5) are present, with the M1 receptor subtype being particularly important for higher-order cognitive processing (Tizabi et al., 2023; Wright et al., 2009).

Under normal conditions, ACh is rapidly degraded in the synaptic cleft by acetylcholinesterase (AChE), which terminates cholinergic signaling. Reductions in ACh levels can arise from decreased synthesis or release, increased AChE activity or expression, or altered cholinergic receptor function (Kuo et al., 2007). In AD, dysfunction of the cholinergic system is well-documented: both the concentration and functional activity of ACh are significantly reduced, impairing cognitive processes (GBD, 2019, 2022). AChE, predominantly located at neuromuscular and neuronal synapses, hydrolyses into acetic acid and choline (Trang and Khandhar, 2023). Its upregulation accelerates ACh breakdown, contributing to synaptic failure and cognitive impairment (Iliyasu et al., 2023).

Notably, AChE also enhances Aβ aggregation by binding to Aβ and inducing fibril formation, generating a neurotoxic Aβ-AChE complex that disrupts normal synaptic function, particularly in the hippocampus (Picciotto et al., 2012; Dinama et al., 2010). Elevated AChE around amyloid plaques and neurofibrillary tangles is a characteristic feature of AD, although the mechanistic significance remains unclear (García-Ayllón et al., 2011).

AChE inhibitors remain the primary pharmacological strategy for symptom management (McGleenon et al., 1999). These inhibitors provide two main therapeutic benefits: (i) enhancement of synaptic ACh levels, improving cognitive function through strengthened cholinergic signaling; and (ii) potential long-term neuroprotective effects, including slower cortical atrophy and delayed clinical progression of dementia (Moss, 2020).

Given the role of ACh across sensory, cognitive, and sleep-wake systems (H Ferreira-Vieira et al., 2016), disruptions in cholinergic neurotransmission amplify AD-related deficits. More specifically, Aβ and tau pathology impairs synaptic integrity within the cholinergic pathways, reducing ACh availability and further compromising neuronal communication (H Ferreira-Vieira et al., 2016; Machado et al., 2020). Early cholinergic lesions occur presynaptically and later extend to post-synaptic nicotinic and muscarinic receptors, particularly M1, reinforcing the rationale for targeting the cholinergic system (Klaassens et al., 2019). Moreover, impaired cholinergic function may intensify downstream AD pathology, including tau phosphorylation, neuroinflammation, and neuronal injury (Chen et al., 2022), further accelerating cognitive decline in AD.

### 3.2 Memantine in AD treatment

The glutamatergic hypothesis of AD proposes that dysregulated glutamate signaling contributes to synaptic dysfunction and neurodegeneration. Glutamate, the major excitatory neurotransmitter in the brain, activates NMDA receptors essential for learning, memory; however, chronic overstimulation leads to excitotoxicity and neuronal injury (Świetlik et al., 2022). MEM, an antagonist of extra-synaptic NMDA receptors, is an established therapeutic option and frequently administered alongside AChE inhibitors. Compared with other non-competitive NMDA receptor antagonists, MEM demonstrates superior tolerability and a more favorable pharmacological profile (Lipton and Chen, 2005).

MEM preferentially binds to open NMDA receptor-operated calcium channels, attenuating pathological calcium influx while preserving physiological synaptic transmission (Yiannopou et al., 2020). Through this mechanism, MEM reduces the harmful effects of sustained glutamate elevation and reduces excitotoxic neuronal loss (Folch et al., 2018). Despite these mechanisms, MEM only provides modest symptomatic benefit, may cause side effects, and does not alter disease progression (Reisberg et al., 2003; Alhazmi and Albratty, 2022). This therapeutic limitation highlights the need for innovative interventions capable of targeting underlying pathophysiological processes rather than solely managing symptoms.

### 3.3 Failure of Aβ-based therapeutics

Aβ peptides accumulate in the brain long before clinical symptoms emerge (Hector and Brouillette, 2021; Porsteinsson et al., 2021). Their aggregation disrupts synaptic function, alters membrane dynamics, and induces structural neuronal damage, including dystrophy, synaptic loss, and impaired neurotransmission (Takahashi et al., 2017; Hashimoto et al., 2003; Zempel et al., 2010). Aβ accumulation also contributes to glutamatergic dysfunction and excitotoxicity (Catania et al., 2019; Gong et al., 2018; de Rojas et al., 2021), which further compromises cholinergic signaling and reduces ACh levels (Pars et al., 2013; Wang and Reddy, 2017). Current FDA-approved therapies, including acetylcholinesterase inhibitors and NMDA receptor antagonists, address these downstream neurotransmitter disturbances but only offer limited symptomatic relief and do not meaningfully modify disease trajectory (Pars et al., 2013; Wang and Reddy, 2017; Ruangritchankul et al., 2021).

Although Aβ has long been a key therapeutic target, many Aβ-directed strategies have failed in clinical trials (Cummings J. L. et al., 2022; Cummings J. et al., 2022; Schott et al., 2019; Anderson et al., 2017). These failures may reflect the complexity and multifactorial nature of AD. Interventions are often initiated after extensive Aβ pathology has already developed, reducing the likelihood of therapeutic success (Anderson et al., 2017). Additionally, targeting Aβ alone is insufficient, as AD progression is shaped by interconnected processes such as neuroinflammation, oxidative stress, autophagy dysfunction, synaptic loss, and dysregulated APP processing (Catania et al., 2019; Gong et al., 2018; de Rojas et al., 2021). Collectively, these factors emphasize the need for multi-targeted or combination therapeutic strategies capable of addressing AD’s broad pathological landscape.

Given the substantial time and financial investment required for new drug development, drug repurposing has emerged as a practical approach to accelerating therapeutic discovery. Repurposed drugs bypass early safety and pharmacokinetic stages and offer a more efficient and lower-risk path toward identifying agents with disease-modifying potential. Ultimately, the clinical imperative remains the development of therapies that can meaningfully slow or reverse AD progression, while improving patient quality of life.

## 4 Autophagy dysregulation in AD pathogenesis

Autophagy is a key cellular pathway responsible for removing damaged proteins and organelles through lysosomal processing (Galluzzi et al., 2017). This process is essential for neuronal function and survival, as neurons are post-mitotic and cannot dilute toxic aggregates through cell division. Moreover, the adult brain has limited regenerative capacity, in part, due to inhibitory glial signaling and a restrictive extracellular environment, making efficient autophagy flux critical for maintaining neuronal homeostasis (Evans and Holzbaur, 2020; Suzuki et al., 2017; Fawcett, 2020).

Autophagic is a dynamic, lysosome-driven process that supports not only intracellular quality control but also key neuronal activities, including axonal maintenance, synaptic function, neuronal connectivity, and stem cell development (Stavoe and Holzbaur, 2019; Valencia et al., 2021; B et al., 2014). Impaired autophagy flux is strongly implicated in neurodegenerative diseases, particularly AD, where defective clearance of protein aggregates contributes to synaptic dysfunction, neuronal injury, and cell death (Zhang et al., 2022; Park et al., 2020). Understanding how autophagy becomes dysregulated in AD is therefore central to identifying new therapeutic targets.

Three primary autophagy pathways operate in mammalian cells: microautophagy, chaperone-mediated autophagy (CMA), and macroautophagy (Galluzzi et al., 2017). Microautophagy involves direct lysosomal membrane invagination, while CMA selectively delivers misfolded proteins to lysosomes via chaperone recognition (Tekirdag and Cuervo, 2018). Macroautophagy, the best-characterized pathway, sequesters cytoplasmic components within double-membrane autophagosomes that subsequently fuse with lysosomes for degradation (Gomes et al., 2017). This pathway is particularly important for clearing large structures, such as protein aggregates and dysfunctional mitochondria (Evans and Holzbaur, 2020; Bloemberg and Quadrilatero, 2019).

Although all three pathways contribute to proteostasis, dysfunction of macroautophagy has been most strongly associated with AD pathology (Ntsapi et al., 2018; Klionsky et al., 2021). As a result, therapeutic strategies aimed at restoring autophagic flux have gained substantial interest as potential interventions to mitigate neurodegeneration and promote neuronal resilience in AD.

### 4.1 Mammalian target of rapamycin complex 1 (mTORC1) - dependent autophagy pathway

Autophagy is tightly coordinated by a conserved network of autophagy-related genes (ATGs) (Levine and Kroemer, 2019). A central upstream regulator of this process is the mammalian target of rapamycin (mTOR), a serine-threonine protein kinase that forms two major complexes: mTOR complex 1 (mTORC1) and mTOR complex 2 (Saxton and Sabatini, 2017). Among these, mTORC1 is the primary metabolic sensor, integrating inputs related to nutrient availability, growth factors, energy status, chronic stress, oxidative stress, and glucocorticoids (Li and hua, 2019; Rabanal-Ruiz et al., 2017).

mTORC1 negatively regulates autophagy; when activated, it inhibits the initiation of autophagosome formation. Hyperactivation of mTORC1, a hallmark of aging, suppresses autophagy flux, resulting in the progressive accumulation of damaged proteins and organelles (Nixon and Yang, 2012). Age-related increases in mTORC1 signalling, combined with general cellular decline, diminish autophagy efficiency (Zinecker and Simon, 2022; Zhang and Cuervo, 2008; Ntsapi and Loos, 2016). As clearance capacity falters, toxic aggregates and dysfunctional organelles accumulate, driving cellular stress and increasing susceptibility to age-associated pathologies, including neurodegenerative diseases.

### 4.2 An overview of the autophagy process

Upstream regulators such as phosphoinositide 3-kinase (PI3K) and protein kinase B (Akt/PKB) primarily through the PI3K/Akt/mTOR pathway (Rabanal-Ruiz et al., 2017). Inhibition of PI3K, Akt, or mTOR promotes autophagy and enhances Aβ clearance in AD models (Nilsson and Saido, 2014; Nilsson et al., 2013; Nilsson et al., 2014; Nixon, 2017; Nixon, 2007). Reactive oxygen species (ROS) also stimulate autophagy by suppressing Akt/mTORC1 signalling (Kaushal et al., 2019; Kma and Baruah, 2022).

**FIGURE 3:**
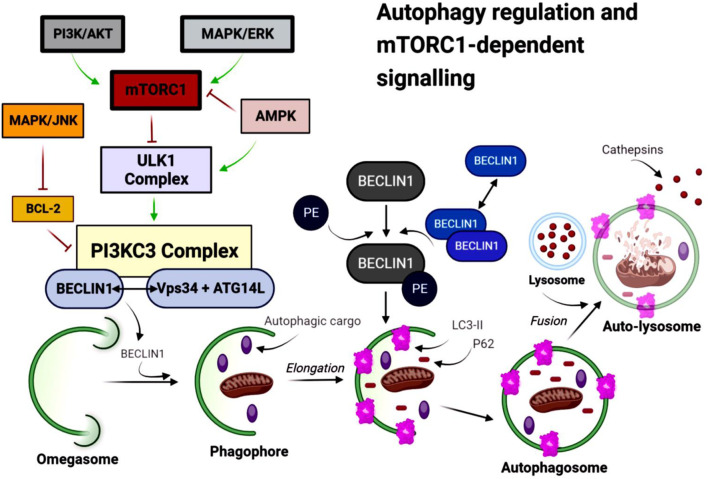
Autophagy regulation by the mTORC1-dependent signalling pathway. Under nutrient-rich conditions, phosphoinositide 3-kinase (PI3K)/AKT signalling activates mTORC1, which inhibits the UNC-51-like kinase 1 (ULK1) complex and suppresses autophagy initiation. Mitogen-activated protein kinase/extracellular signal-regulated kinase (MAPK/ERK) signalling also interacts with mTORC1, enhancing its activity and further contributing to autophagy suppression. In contrast, low adenosine triphosphate (ATP) levels activate AMP-activated protein kinase (AMPK), which inhibits mTORC1 and promotes ULK1 activation. Activated ULK1 stimulates class III phosphatidylinositol 3-kinase complex (PI3KC3; Vps34-ATG14L-Beclin 1), driving omegasome formation and phagophore nucleation. Beclin 1 is essential for omegasome nucleation and facilitates microtubule-associated protein 1 light chain 3 (LC3) lipidation. LC3 is conjugated to phosphatidylethanolamine (PE) to form LC3-II, a modification coordinated by Beclin 1 that enables phagophore elongation and autophagosome maturation. MAPK/c-Jun N-terminal kinase (MAPK/JNK) signalling inhibits B-cell lymphoma 2 (Bcl-2), relieving its suppression of the PI3KC3-Beclin 1 complex and thereby promoting autophagy initiation. The selective autophagy receptor sequestosome-1 (p62/SQSTM1) binds ubiquitinated cargo and is sequestered into the expanding phagophore. Mature autophagosomes fuse with lysosomes to form autolysosomes (autophagolysosomes), where autophagic cargo is degraded by lysosomal hydrolases and recycled into amino acids.

Under nutrient-rich conditions, active mTORC1 inhibits autophagy initiation by phosphorylating the Unc-51-like autophagy activating kinase 1 (ULK1) complex (Rabanal-Ruiz et al., 2017). Upon mTORC1 inactivation, the ULK1 complex triggers formation of the phagophore, the precursor membrane that expands to form the autophagosome. The phagophore elongates through two ubiquitin-like conjugating systems: the ATG12-ATG5-ATG16L complex and microtubule-associated protein light chain 3 (LC3I, MAP1LC3B) (Galluzzi et al., 2017; Yang and Klionsky, 2010). LC3 is converted from its cytosolic (LC3-I) to its lipidated, membrane-bound form (LC3-II), which stabilizes the autophagosomal membrane and facilitates fusion with lysosomes (Wang et al., 2013) (Figure 3). Autophagosomes sequester cytoplasmic cargo, including APP, Aβ, and tau-containing aggregates, relevant to AD pathology (Lucà et al., 2021; Yu and Goncharova, 2022; Harrison et al., 2009).

Mature autophagosomes fuse with lysosomes to form an autophagolysosome (autolysosome), where cargo is degraded by lysosomal hydrolases and the resulting metabolites are recycled (Suzuki et al., 2017; Yang and Klionsky, 2010). LC3-II serves as a marker of autophagosome formation, whereas decreased levels of sequestosome 1 (p62/SQSTM1) indicate efficient autophagic flux (B et al., 2014).

Beyond mTORC1, AMP-activated protein kinase (AMPK) is a key metabolic regulator that promotes autophagy under low-energy conditions (Li and Chen, 2019; Wang S. et al., 2022; Tamargo-Gómez and Mariño, 2018). AMPK both inhibits mTORC1 and directly activates ULK1 (Wang S. et al., 2022; Garcia and Shaw, 2017). It also regulates the class III phosphatidylinositol 3-phosphate (class III PI3P) complex - comprising vacuolar protein sorting 34 (Vps34), Beclin-1 (BECN1), and Atg14L - which drives autophagosome nucleation (Suzuki et al., 2017; Menon and Dhamija, 2018; McKnight and Zhenyu, 2013).

mTORC2 also contributes to autophagy regulation by phosphorylating Akt (protein kinase B, PKB), which in turn enhances mTORC1 signaling (Gao et al., 2014). Full activation of mTORC1additionally requires the small GTPase Rheb, which is controlled by the tuberous sclerosis complex subunit 2 (TSC1/2) (Lemmon and Schlessinger, 2010; Khan et al., 2013). Akt suppresses TSC1/2, thereby releasing Rheb and promoting mTORC1 activation (Ntsapi et al., 2018; Zou et al., 2020; Lucà et al., 2021). Dysregulation of both mTORC1 and mTORC2 has been implicated in AD (Yu and Goncharova, 2022; Boutouja et al., 2019), with reduced levels of each complex observed in human AD brain tissue (Lee et al., 2017). Pharmacological modulation of mTORC1 restores autophagic balance and demonstrates therapeutic potential in transgenic AD models (Lee et al., 2017; Harrison et al., 2009; Carro et al., 2006).

### 4.3 Impaired autophagy and protein aggregation in AD

In AD, the accumulation of Aβ compromises autophagy at later stages of the pathway, contributing to neuronal dysfunction and accelerated neuronal death *in vitro* and *in vivo*, as reported by Ntsapi and Loos (Ntsapi and Loos, 2021) and Lee et al. (Lee et al., 2022). Aβ accumulation is the central hallmark of AD and exerts neurotoxic effects. Dysregulated or suboptimal autophagy activity is therefore considered a key contributor to the onset and progression of AD (Cai et al., 2015). Whether autophagy is protective or harmful depends on the context, including the type of stress, the duration and intensity of autophagy induction, the efficiency of lysosomal clearance, and the overall “autophagic flux” (Jaeger and Wyss-Coray, 2009). Deleterious overactivation or prolonged induction may lead to autophagic cell death, occurring when autophagy persists excessively, causing the degradation of cytoplasm, organelles, or essential components, leading to energy failure and neuronal death. In contrast, neuroprotective autophagy typically occurs under normal or moderately stressed conditions, acting as a quality control mechanism that removes toxic proteins, maintains metabolic and organelle homeostasis, reduces neurotoxicity, and supports neuronal health (Cui and Zhao, 2025; Son et al., 2012).

Increased mTORC1 activity, associated with aging, further suppresses autophagy and promotes the buildup of damaged proteins and organelles (Nixon and Yang, 2012; Nixon and Yang, 2011). Age-related decline in autophagic efficiency is thus thought to facilitate the pathological processes underlying AD (Aman et al., 2021).

Autophagy is the primary intracellular mechanism involved in modulating APP processing and Aβ production, with substantial amounts of APP and its cleavage products found within autophagic vacuoles (AVs) (Nixon et al., 2005). AVs refer broadly to vesicular structures engaged in the autophagy pathway, including the pre-autophagosomal structures, autophagosomes, lysosomes, or autophagolysosomes (Nixon, 2017; Boland et al., 2008). These vesicles contain immunoreactive Aβ and APP-related cleavage products *in vivo* AD models (Nixon, 2007), highlighting their central role in Aβ generation and clearance.

Inhibition of mTOR has been shown to induce autophagy and reduce BACE expression in the APP/PS1 transgenic mouse model of AD (Nilsson et al., 2014; Nilsson et al., 2014; Rahman et al., 2020). Conversely, the autophagy inhibitor, 3-methyladenine (3-MA), enhances γ-secretase activity and increases Aβ production (Park et al., 2020), highlighting the importance of maintaining normal autophagy activity for APP clearance. Loss of key ATGs leads to neurodegeneration driven by the accumulation of ubiquitinated aggregates (Karsli-Uzunbas et al., 2014; Kim et al., 2015; Jogalekar et al., 2021; Towers and Thorburn, 2016), whereas enhanced autophagy flux reverses protein buildup and supports neuronal and organismal health (Suresh et al., 2020).

Excessive activation of BACE and γ-secretase accelerates AD progression by increasing APP processing and Aβ formation (Sinha et al., 1999; Vassar et al., 1999), further underscoring the regulatory role of autophagy in APP metabolism (Rahman et al., 2020; Kuang et al., 2020). Pharmacological induction of autophagy in animal models of AD promotes early degradation of APP and reduces (Tian et al., 2011). Ntsapi and Loos (Ntsapi and Loos, 2021) reported that the persistent Aβ accumulation compromises autophagy at later stages, leading to neuronal dysfunction and accelerated cell death *in vitro*. Indeed, dysregulated or insufficient autophagy is considered a major contributor to AD development and progression (Ntsapi et al., 2018; Nixon et al., 2005; Suresh et al., 2020).

Autophagy is also central to the intracellular processing of APP and Aβ, as AVs contain high levels of APP and its cleavage products (Nilsson et al., 2014; Nixon, 2007; Bordi et al., 2016). AVs in AD models contain immunoreactive Aβ and APP precursors (Ntsapi and Loos, 2016; Lemmon and Schlessinger, 2010), and the relatively stable levels of APP within AVs indicate that the cleavage of APP can also occur inside these vesicles (Nixon, 2007). This suggests that AVs may serve as sites for abnormal APP processing (Yu et al., 2004). Consistent with these findings, inhibition of mTOR induces autophagy and reduces BACE expression in the APP/PS1 transgenic mouse model of AD (Nilsson et al., 2013; Nilsson et al., 2014; Nixon et al., 2005). In contrast, the autophagy inhibitor 3-MA enhances γ-secretase activity and increases Aβ production (Cai et al., 2015; Yu et al., 2004). Taken together, maintaining functional autophagy is essential for efficient and sustained clearance of APP and Aβ-related substrates in neuronal cells.

### 4.4 Modulation of autophagy in AD

There is substantial evidence supporting the therapeutic potential of targeting autophagy in the treatment of AD (Zhao et al., 2020; Aman et al., 2021; Li et al., 2022). In AD, impaired autophagy and protein aggregation reinforce one another in a vicious cycle that accelerates neurodegeneration, highlighting the need to identify safe and effective autophagy modulators. Recent research has explored cannabinoid-based therapeutics, which have shown promise in inducing autophagy and promoting cell death *in vivo* and *in vitro* in the context of cancer (Lastres-Becker et al., 2005; García et al., 2011). For example, Salazar and colleagues demonstrated that activation of the autophagy pathway is required for the antitumor effects of cannabinoids (Salazar et al., 2009). Additional work in MDA-MB-231 breast cancer cells indicated that cannabinoid-induced cell death and autophagy can occur independently of cannabinoid receptor activation. More recently, cannabidiol was shown to induce autophagy in an *in vitro* model of PD (Kang et al., 2021).

Collectively, these studies suggest that cannabinoid-based treatment approaches may hold therapeutic potential for a range of human diseases, including AD. However, further research is needed to identify cannabinoid-derived modulators that are both safe and effective, and to clarify the mechanisms through which cannabinoid-based autophagy may contribute to AD treatment. Continued advances in understanding pharmacokinetics and neuronal effects of cannabinoid-based therapeutics have made this an increasingly prominent area of biomedical research.

## 5 The endocannabinoid system

**FIGURE 4:**
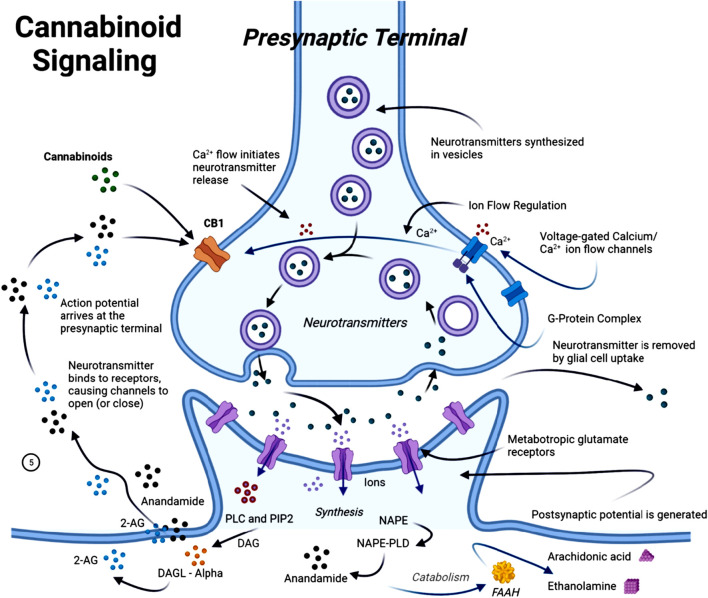
Simplified scheme representing endocannabinoid system-modulated synaptic transmission. The endocannabinoids anandamide (AEA) and 2-arachidonoylglycerol (2-AG) are not stored in vesicles but are synthesized *de novo* from phospholipid precursors through calcium-dependent mechanisms. N-acylphosphatidylethanolamine (NAPE) is hydrolyzed by N-acylphosphatidylethanolamine-specific phospholipase D (NPLD) to yield AEA, while diacylglycerol (DAG) is converted to 2-AG by diacylglycerol lipase (DAGL). These lipid messengers diffuse retrogradely across the synaptic cleft to activate presynaptic cannabinoid receptor type 1 (CB1). CB1 receptor activation modulates ion channels, inhibits voltage-gated calcium influx, and suppresses neurotransmitter release, thereby providing negative feedback to regulate synaptic strength. Endocannabinoid signalling is terminated by enzymatic degradation: AEA is primarily metabolized to arachidonic acid (AA) and ethanolamine by fatty acid amide hydrolase (FAAH) in the postsynaptic neuron, whereas 2-AG is hydrolyzed presynaptically into AA and glycerol by monoacylglycerol lipase (MAGL). Through these mechanisms, the endocannabinoid system modulates synaptic strength, neuronal excitability, and overall network homeostasis.

The endocannabinoid system (ECS) is a complex cell-signalling system in humans and other animals (Yoder and Watson, 2017). It regulates a wide range of physiological and cognitive processes, including motor function (Young and Denovan-Wright, 2022; Basavarajappa et al., 2017), synaptic plasticity (Piette et al., 2020; Heifets and Castillo, 2009), neuroinflammation (Burstein and Zurier, 2009; Marchalant et al., 2009), and neural cell fate (Guzmán et al., 2002; Guzmán, 2003). The medical benefits of the *Cannabis sativa* plant date back to ancient times, with the earliest documented references appearing in the “Pen-ts’ao ching”, regarded as the world’s oldest *pharmacopeia* (China, 2737 BC) (Pantoja-Ruiz et al., 2022; Zuardi, 1999). In recent years, research interest has increasingly shifted toward targeting the ECS and investigating the use of phytocannabinoids, naturally occurring compounds that are found in the *Cannabis sativa* plant, as a potential therapeutic agent. This interest is largely driven by growing evidence of ECS dysfunction during the progression of AD (Núñez et al., 2004; Westlake et al., 1994; Benito et al., 2008; Russo, 2018). The ECS plays a central role in maintaining homeostasis across several physiological functions, including cognition, anxiety regulation, pain perception, neurogenesis, immune signalling, inflammation, and synaptic responsiveness and plasticity (Chen et al., 2012). The ECS consists of two primary G-protein-coupled receptors, cannabinoid receptors 1 and 2 (CB1R and CB2R), as well as endocannabinoids such as anandamide (AEA) and 2-arachidonoylglycerol. It also includes metabolic enzymes such as fatty acid amide hydrolase (FAAH) and monoglyceride lipase (MAGL), which are responsible for the synthesis and degradation of endocannabinoids (Alexander, 2016; Aizpurua-Olaizola et al., 2017) (Figure 4).

### 5.1 The effects of cannabinoids on autophagy activity

Within the nervous system, the ECS is composed of naturally occurring lipid signalling pathways involved in numerous neurophysiological processes (Schuster et al., 2015). This system consists of endocannabinoid receptors, primarily CB1, predominantly expressed in the CNS, and CB2, mainly expressed peripherally in the microcirculation, along with metabolic enzymes that regulate the synthesis and degradation of endocannabinoids (Vrechi et al., 2021; Wang Z. et al., 2022). Through these components, the ECS plays a central role in maintaining neuronal function and homeostasis (Ghosh et al., 2021).

Autophagy is an evolutionarily conserved cellular degradation process that maintains cellular homeostasis and responds to proapoptotic stimuli and programmed cell death. It relies on the formation of specialized structures, including phagosomes, autophagosomes, and autophagolysosomes (Padman et al., 2019). Dysregulated autophagy has been implicated in a range of human diseases, including neurodegenerative disorders, and is closely linked to inflammatory processes (Ghosh et al., 2021; Chung et al., 2017). Autophagy can interact with apoptosis pathways, either promoting cell survival by downregulating apoptosis or contributing to cell death when co-activated with apoptotic mechanisms (Salazar et al., 2009; Lu et al., 2019).

Cannabinoids, particularly cannabidiol (CBD and tetrahydrocannabinol (THC), exhibit neuroprotective and anti-inflammatory effects, partly through autophagy modulation. One possible mechanism by which cannabinoids exert anti-inflammatory and neuroprotective effects is through the regulation of mitochondrial function through autophagy (Lu et al., 2019; Zhou et al., 2011; Ji et al., 2021). They can regulate mitochondrial function and activate peroxisome proliferator-activated receptor (PPAR)γ, restoring autophagy, modulating apoptosis, and enhancing mitochondrial β-oxidation and biosynthesis (O’Sullivan, 2016; Ni et al., 2022; Ribeiro et al., 2012). Activation of PPAR by cannabinoids has been associated with reduced inflammation in models of acute and chronic neuronal injury (Kapadia et al., 2008).

In animal studies, chronic administration of THC has been shown to influence apoptosis and immune responses in animal models, promoting cell survival and reducing inflammatory damage (Molina et al., 2014).

Notably, cannabinoids modulate key autophagy-regulatory pathways. CBD inhibits phosphorylation of PI3K, Akt, and mTORC1 in human cholangiocarcinoma cells, increasing LC3B-II and decreasing p62 expression–classical markers of enhanced autophagic flux (Pongking et al., 2024). Although this study used a cancer model, it demonstrates that cannabinoids can shift PI3K/Akt/mTOR signalling toward autophagy, rather than proliferation. In neuronal models, CBD induces autophagy via Akt and extracellular signal-regulated kinase (ERK) modulation, independently of mTOR (Vrechi et al., 2021). Using human SH-SY5Y neuroblastoma cells, Vrechi et al. (2021) reported that 10 µM CBD increased autophagic flux (LC3-II, autophagosome/lysosome formation), reduced Akt phosphorylation, and activated ERK1/2, without altering classical mTORC1 downstream targets, indicating an mTOR-independent, ULK1-dependent mechanism. CBD also enhances neuronal health and longevity via autophagy. In hippocampal neurons, SH-SY5Y cells, and *in vivo* (*Caenorhabditis elegans*), CBD promoted autophagic flux, improved neuronal integrity, these effects were dependent on core ATGs and the sirtuin pathway, suggesting a multi-pathway mechanism converging on proteostasis and neuroprotection (Wang Z. et al., 2022).

Collectively, these findings indicate that cannabinoids can modulate autophagy through both PI3K/Akt/mTOR-dependent and independent mechanisms, enhancing neuronal resilience, maintaining proteostasis, and potentially counteracting neurodegenerative processes.

### 5.2 Cannabinoid-induced autophagy: receptor-dependent and independent pathways

Cannabinoid-induced autophagy operates through both receptor-dependent and receptor-independent mechanisms, varying with cell type and signalling context. In human SH-SY5Y neuroblastoma cells and murine astrocytes, CBD-induced autophagic flux was markedly reduced by antagonists of CB1, CB2, and transient receptor potential vanilloid 1 (TRPV1) - a Ca^2+^-permeable ion channel that drives autophagy via Ca^2+^/AMPK and ERK pathways (Karki et al., 2015). This indicates that activation of these receptors contributes to CBD’s autophagy-modulating effects. CBD also modulated signalling pathways by suppressing Akt phosphorylation and activating ERK1/2, with autophagy induction dependent on ULK1, but independent of classical mTORC1 downstream targets (Vrechi et al., 2021). These observations suggest a receptor-dependent initiation step that feeds into a non-classical, mTOR-independent autophagy mechanism in neuronal cells.

Conversely, there is also evidence that supports receptor-independent activation of autophagy by cannabinoids. In cancer cell models, THC and related cannabinoids induce autophagy and cell death through endoplasmic reticulum (ER) stress, ceramide accumulation, and phosphorylation of eukaryotic initiation factor 2α, leading to inhibition of Akt/mTORC1 signalling and autophagy induction independent of cannabinoid receptor activation (Salazar et al., 2009). Cannabinoids can also trigger autophagy through non-receptor mechanisms such as ROS generation, ER stress, and activation of transient receptor potential channels, a family of calcium-permeable ion channels involved in Ca^2+^-dependent autophagy signalling (Oakes et al., 2019). These findings support a broader ligand–receptor-independent network through which cannabinoids can modulate autophagy. Taken together, current evidence supports a unified model in which cannabinoids induce autophagy through both receptor-dependent and receptor-independent pathways. In receptor-expressing cells, cannabinoids activate canonical targets (CB1/CB2/TRPV1), triggering downstream signalling (e.g., ERK1/2 activation, Akt inhibition) that engages ULK1 and initiates autophagy, often independent of mTORC1 regulation. In other cellular contexts - particularly stressed, non-neuronal, or transformed cells - cannabinoids can bypass surface receptors, promoting ER stress, ceramide accumulation, or ROS generation that converge on autophagic induction via suppression of survival pathways or activation of stress-response signalling. These two complementary mechanisms help reconcile divergent findings across models and demonstrate that cannabinoid-induced autophagy is context-dependent and driven by partially overlapping pathways. In neurodegenerative settings, such flexibility may be beneficial, as non-canonical pathways could maintain pro-autophagic activity even when receptor expression is altered (e.g., CB1 downregulation), thereby supporting proteostasis and cellular clearance.

### 5.3 ECS alterations in AD: implications for therapeutic strategies

The ECS has demonstrated neuroprotective properties against excitotoxicity, inflammation, and oxidative stress - pathological processes central to AD and targets of many current AD therapies (Xiong and Lim, 2021; van der Stelt et al., 2006; Trezza and Campolongo, 2013). Early evidence for a role of the ECS’s in AD was based on the high density of CB1 receptors in the hippocampus and cerebral cortex (Riedel and Davies, 2005), as well as an increased expression of the endocannabinoid-metabolizing enzyme, FAAH, on plaque-associated astrocytes (Benito et al., 2003).

AD is associated with ECS alterations, though it remains debated whether ECS upregulation serves to counteract neuronal hyperactivity and neuroinflammation or whether it contributes to symptoms such as memory loss (O’Sullivan, 2016; Ni et al., 2022). Consistently, studies have observed increased CB2R receptor expression in the brain tissue of individuals with AD and in AD-relevant animal models. This upregulation reflects immunomodulatory responses to pathogenic events. For example, clinical studies report elevated CB2R expression in microglia near hippocampal plaques (Ji et al., 2021), with a positive correlation between CB2R expression and Aβ42 levels and plaque burden (Ribeiro et al., 2012). Similarly, rats treated with Aβ exhibit increased CB2R levels (Monteiro et al., 2020; Esposito et al., 2006). Activation of microglial CB2R promotes Aβ clearance in human AD tissue sections (Tolón et al., 2009), and prevents Aβ-induced microglia activation *in vitro* and *in vivo* (Ramírez et al., 2005).

In contrast, reductions in CB1R receptor expression have been observed in areas of microglial activation in both humans and AD-relevant rodent models (Ramírez et al., 2005), and decreases in CB1R and the endocannabinoid AEA were reported in Aβ-treated rats (Esposito et al., 2007a). Moreover, cortical downregulation of AEA in AD post-mortem tissue correlates inversely with Aβ42 levels and cognitive deficits (Jung et al., 2012).

The ECS holds therapeutic potential due to its broad neuromodulatory effects, which in some cases mimic the results of FDA-approved drugs or provide superior symptom relief. For example, the synthetic endocannabinoid HU-211 (Dexanabinol), acts as a stereoselective NMDA receptor inhibitor, similar to MEM, reducing calcium influx and limiting excitotoxicity (Gowran et al., 2011; Nadler et al., 1993). Endocannabinoid-mediated neuroprotection against excitotoxicity is a well-established and can occur through multiple mechanisms (Kasatkina et al., 2021; Xu et al., 2017; Aguado et al., 2007; Wu et al., 2019). While the AD-ECS relationship is complex and requires further investigation, these findings suggest that key ECS components are critically involved in AD pathology and represent promising therapeutic targets.

## 6 The effects of phytocannabinoids on Aβ-related pathology in AD

Cannabis sativa (commonly known as hemp or marijuana) is a multipurpose plant belonging to the family Cannabaceae, widely used for medicinal, recreational, and industrial purposes (Dowling et al., 2021). The plant contains more than 500 bioactive compounds, including over 100 phytocannabinoids, with diverse pharmacological properties (Gülck and Møller, 2020; Caprioglio et al., 2022). These compounds have attracted significant interest for their potential therapeutic effects in neurodegenerative diseases such as AD.

Among them, THC and CBD are the most extensively studied for their neuroprotective and disease-modifying potential (Elsohly and Slade, 2005; Reekie et al., 2017). Although they share the same molecular formula (C_21_H_30_O_2_) and a terpenophenolic backbone, their distinct functional groups result in different receptor interactions and pharmacokinetic profiles. THC contains a cyclic ether (closed-ring) structure and acts primarily as a partial agonist at CB_1_ receptors within the CNS. In contrast, CBD contains free hydroxyl groups (open-chain), shows low direct affinity for CB_1_ receptors, and instead modulates the ECS indirectly through allosteric and non-canonical pathways (Pertwee, 2008).

Both CBD (non-intoxicating) and THC modulate the ECS and exhibit neuroprotective, anti-inflammatory, and antioxidant properties. Emerging evidence suggests that these phytocannabinoids exert therapeutic-like effects on AD-related pathology, including Aβ accumulation and tau hyperphosphorylation (Coles et al., 2022; Barber et al., 2023; Libro et al., 2016). Collectively, these findings highlight the therapeutic promise of cannabinoids as modulators of key neurodegenerative pathways and support further investigation into their molecular mechanisms and translational potential for AD management.

### 6.1 CBD’s neuroprotective potential in AD

Preclinical evidence increasingly supports the therapeutic potential of CBD in AD through multiple molecular and cellular mechanisms.

*In vitro,* CBD demonstrates several neuroprotective properties directly relevant to AD pathology. It inhibits tau hyperphosphorylation and reduces APP expression in APP-transfected human neuroblastoma cells. CBD also downregulates BACE1, PS1, and PS2 - key genes involved in the enzymatic generation of Aβ (Libro et al., 2016). Additionally, CBD protects against Aβ-induced cytotoxicity in inducible human neuron-like cell models (Schubert et al., 2019), while hemp CBD-rich hemp seed oil (∼80% CBD) significantly reduces Aβ_42_ + Cu(II)-induced oxidative stress (Raja et al., 2020). Beyond its antioxidant effects, CBD has been shown to restore mitochondrial function (Raja et al., 2020; Esposito et al., 2007b), a central feature of AD pathogenesis. Recent preclinical findings further reinforce these mechanisms, showing that CBD decreases phosphorylated tau and Aβ aggregation, reduces their axonal spread between cortical and hippocampal neurons, and promotes microglial polarization toward a neuroprotective M2 phenotype (Raïch et al., 2025). This study additionally showed that CBD reduces ROS formation, enhances neuronal viability, and partially restores neurite formation disrupted by Aβ and Tau, highlighting CBD’s role in supporting neuronal structural integrity.

*In vivo* studies further support these neuroprotective effects. Short-term CBD treatment (7 days) attenuates Aβ-evoked neuroinflammation (Martín-Moreno et al., 2011), whereas chronic administration of 20 mg/kg CBD prevents learning and memory impairments in Aβ pharmacological mouse models (Coles et al., 2022; Cheng et al., 2014a). Dose-dependent effects have also been observed in APP/PS1 transgenic mice, where chronic CBD treatment (5–50 mg/kg) not only reverses established cognitive deficits and, at 20 mg/kg, also prevents their onset (Coles et al., 2022; Cheng et al., 2014b; Cao et al., 2014). These results align with emerging evidence showing that chronic daily CBD administration (10 mg/kg for 28 days) significantly improves both short- and long-term spatial memory in 5xFAD mice, underscoring CBD’s capacity to ameliorate behavioral and cognitive impairments in AD models (Raïch et al., 2025). Additional evidence from a sporadic AD model demonstrates that CBD restores object recognition, spatial memory, and social behavior, reduces hippocampal pro-inflammatory gene expression, mitigates increases in Aβ and p-Tau, and exerts its cognitive and anti-inflammatory effects predominantly through CB1 receptor activation (Toledano and Akirav, 2025).

Although clinical data remain limited, expanding translational interest has triggered several ongoing clinical trials investigating CBD-based therapies for AD (e.g., NCT04436081, 2019-002106-52, ACTRN12621001364864). Notably, emerging evidence from a 26-week randomized controlled trial of low-dose THC-CBD extract reported significantly improved MMSE scores and a favorable safety profile compared with placebo (Raïch et al., 2025).

Collectively, the preclinical evidence highlights CBD’s multimodal actions, including anti-inflammatory, antioxidant, mitochondrial-restorative, and anti-amyloidogenic, underscoring its promise as a disease-modifying candidate for AD intervention.

### 6.2 THC’s neuroprotective potential in AD

THC is the principal psychoactive phytocannabinoid in *Cannabis sativa*. Structurally, it is a diterpenoid featuring a 6a,7,8,10a-tetrahydro-6H-benzo [c]chromene core substituted by a hydroxyl group at position 1, two methyl groups at position 6, one methyl group at position 9, and a pentyl group at position 3 (Tyrakis et al., 2024). Pharmacologically, THC acts primarily as a partial agonist at CB_1_ and CB_2_ receptors, producing neuroactive, analgesic, and psychotropic effects (Lee et al., 2024; Zou and Kumar, 2018; Paronis et al., 2012). Its actions extend beyond canonical ECS to include modulation of neurotransmission, oxidative stress, and amyloid processing—mechanisms highly relevant to AD pathophysiology.

Preclinical evidence indicates that THC targets multiple AD-related pathways. *In vitro*, THC attenuates pathological processes in neuro-2a APPSwe cells (Marsh et al., 2024). A notable finding is its competitive inhibition of AChE, preventing the breakdown of ACh, a neurotransmitter significantly depleted in AD (Sam and Bordoni, 2023; Puopolo et al., 2022). By enhancing cholinergic signalling, THC may provide symptomatic benefits similar to conventional AChE inhibitors but without the hepatotoxicity associated with agents such as Tacrine (Cognex). THC has also been shown to inhibit Aβ aggregation, thereby potentially reducing amyloid plaque formation and associated neurotoxicity (Eubanks et al., 2006; Calabrese and Rubio-Casillas, 2018).

Recent preclinical findings further reinforce THC’s mechanistic relevance in AD models. A 2024 study using APP/PS1 mice demonstrated that low-dose THC combined with CBD modulates hippocampal glutamate dynamics, reducing extracellular glutamate and hippocampal hyperexcitability - a key contributor to excitotoxic injury in AD (Marsh et al., 2024). Complementing these *in vivo* findings, the *in vitro* experiments using cannabis extracts standardized for THC demonstrated neuroprotection against Aβ-induced cytotoxicity, improving neuronal viability and reducing amyloid-mediated oxidative damage.

In clinical contexts, THC-based formulations have primarily been used for appetite stimulation, antiemetic purposes, and the management of behavioral and psychological symptoms of dementia, including agitation and weight loss (223–226). Such interactions are dose-dependent and often biphasic, meaning that therapeutic outcomes depend critically on dose ratios, treatment duration, and biological context (232–234).

Although clinical evidence remains limited, research interest in THC-based interventions for AD is increasing. Two ongoing clinical trials (NCT04516057, NCT02792257) are specifically assessing THC-containing treatments for agitation in AD patients. These studies aim to determine the optimal dosing strategies, safety profiles, and whether THC - alone or in combination with CBD - may offer symptomatic relief or even disease-modifying potential. These investigations are supported by preclinical and narrative review evidence, which highlights THC’s multilayered neuroprotective effects, including anti-inflammatory, antioxidant, and anti-amyloidogenic actions (Tyrakis et al., 2024), positioning it as a promising candidate for modulating AD-related neurodegeneration.

### 6.3 Multi-cannabinoid approaches in AD pathophysiology

Although clinical evidence remains limited, research interest in cannabinoid-based interventions for AD has increased substantially in recent years. Two ongoing trials (NCT04516057, NCT02792257) are evaluating THC-containing interventions for agitation in AD patients, reflecting a broader shift toward assessing cannabinoids for both behavioral and potential neuroprotective effects. Emerging evidence increasingly supports multi-cannabinoid formulations, particularly THC and CBD combinations, which produce effects not observed with either compound alone.

The first robust clinical evidence for such combinations was provided by Cury et al. (2025), who conducted a 26-week, randomized, double-blind, placebo-controlled Phase-2 trial of a low-dose balanced THC: CBD extract (0.350 mg THC +0.245 mg CBD daily) in older adults with AD-associated dementia. The THC: CBD group showed significant improvement in Mini-Mental State Examination (MMSE) scores versus placebo, without increased adverse events (Cury et al., 2025). Although secondary outcomes were unchanged, the study demonstrates that chronic low-dose THC: CBD can be safely administered and provides preliminary evidence of symptomatic cognitive benefit, warranting larger, biomarker-rich trials.

Mechanistic is provided by Sánchez-Fernández et al. (2024), who examined hippocampal glutamate dynamics in APP/PS1 mice chronically treated with non-psychoactive doses of THC + CBD. The combination, but not either cannabinoid alone, reduced extracellular glutamate levels and lowered hippocampal excitability, two alterations closely linked to excitotoxic injury and cognitive decline in AD. Notably, these effects occurred without overt changes in synaptic structure or canonical synaptic plasticity markers, suggesting that THC + CBD may act through modulation of glutamate uptake, clearance, or network-level excitability rather than direct synaptic remodelling (Sánchez-Fernández et al., 2024). These results support a mechanistic rationale for combination therapy, with THC and CBD appearing to produce emergent effects that cannot be inferred from single-approach studies. This highlights the importance of including neurochemical and electrophysiological biomarkers, such as magnetic resonance spectroscopy (glutamate), EEG markers of excitability, and fluid synaptic injury markers, when evaluating cannabinoid interventions in translational studies.

Further preclinical evidence from Arnanz et al. (2024) in the 5xFAD mouse model, an aggressive amyloidogenic AD model, showed that chronic low-dose treatment with a THC: CBD combination improved spatial memory, whereas the single agents did not. Interestingly, all cannabinoid-treated groups, including the combination, exhibited increased Aβ_42_ cortical levels, highlighting complex effects on amyloid processing. Cognitive improvements occurred without significant changes in classical inflammatory markers, underscoring that multi-cannabinoid therapy may exert functional benefits via mechanisms distinct from classical anti-inflammatory pathways. These findings indicate that while combination therapy offers synergistic behavioural benefits, careful longitudinal assessment of amyloid and other disease-modifying endpoints is essential.

Collectively, these studies highlight that multi-cannabinoid regimens exert synergistic effects across cognitive, excitotoxic, and network-level outcomes. THC and CBD engage complementary mechanisms, including CB1/CB2 modulation, glutamate regulation, and antioxidant activity, while divergent effects on amyloid pathology underscore the need for mechanistically informed, biomarker-guided clinical trials. Overall, the evidence provides a strong rationale for advancing multi-cannabinoid therapeutics in AD, with clear priorities for dose optimization, biomarker integration, and longitudinal evaluation to determine both symptomatic and disease-modifying potential in future translational and clinical studies.

### 6.4 Potential cannabinoid interactions with FDA-approved AD therapies

Multi-cannabinoid interventions in AD raise important considerations regarding interactions with FDA-approved therapies, including AChEIs and MEM. Recent *in vitro* studies demonstrate that CBD, THC, and other cannabinoids moderately inhibit AChE and butyrylcholinesterase (BChE), a related cholinergic enzyme that hydrolyzes acetylcholine and other choline esters. In AD, BChE activity increases in later stages, and its inhibition can help sustain cholinergic signaling, complementing AChE-targeted therapies (Puopolo et al., 2022; Patil et al., 2023). While these interactions suggest potential synergy, high doses could theoretically amplify cholinergic effects and increase adverse events.

Interactions with MEM are more complex: both cannabinoids and MEM modulate glutamatergic signaling, with preclinical data indicating potential for synergistic neuroprotection as well as receptor-level antagonism depending on dose and context (Marszalek-Grabska et al., 2021). Pharmacokinetic interactions are also notable, as CBD and THC influence cytochrome P450 enzymes (CYP3A4, CYP2C19, CYP2D6), which metabolize many AChEIs and memantine, highlighting the need for dose optimization and careful monitoring in older adults with polypharmacy (Balachandran et al., 2021; Beers et al., 2021).

Future studies should prioritize preclinical combination models alongside early-phase clinical trials to comprehensively evaluate safety, pharmacokinetics, pharmacodynamics, and efficacy. This includes preclinical experiments assessing cognitive, synaptic, and biomarker outcomes, as well as phase 1-2 trials examining cannabinoids in combination with AChEIs or MEM. Such research is essential to determine whether multi-cannabinoid therapy can safely and effectively complement existing AD treatments, optimizing symptomatic relief while minimizing potential adverse interactions.

## 7 Conclusion

The therapeutic potential of cannabinoid-based interventions for AD is well-supported; however, the inherent chemical complexity of *Cannabis sativa* continues to pose significant translational challenges. Precisely defined formulations, as well as careful control of dose, ratio, and route of administration routes, remain essential to achieving consistent therapeutic outcomes. Emerging evidence, including a recent case report (Ruver-Martins et al., 2022), suggests that cannabinoid microdosing may offer a potential strategy for reducing AD-related symptoms while minimizing adverse effects. Yet, these preliminary findings require rigorous validation through larger, well-controlled clinical studies. Pharmacokinetic data further indicate that multi-cannabinoid formulations, particularly those combining THC and CBD, add additional minor cannabinoids, may provide enhanced therapeutic efficacy and improved safety profiles compared to monotherapy (Zuardi et al., 2012). However, it is important to note that evidence on cannabinoid-induced autophagy in AD remains limited and heterogeneous. Most studies are preclinical, with variations in cannabinoid type, dose, and measurement of autophagy markers, and quantitative data in disease-relevant models are sparse, highlighting a critical knowledge gap. Although both *in vitro* and *in vivo* studies increasingly support the potential of such combination therapies, the mechanistic interplay between multi-cannabinoid therapeutics, autophagy modulation, and neuronal survival remains insufficiently understood. Therefore, there is an urgent need for comprehensive, mechanistically grounded research to define the optimal THC: CBD ratio and broader cannabinoid profiles capable of effectively modulating autophagy, mitigating amyloidogenic and inflammatory pathways, and ultimately providing robust neuroprotection in AD-relevant experimental systems. Such work will be critical for translating cannabinoid-based treatments from promising preclinical leads into safe, targeted, and effective clinical interventions for AD.

## Glossary

AD: Alzheimer’s disease
APP: Amyloid precursor protein
PS1/2: Presenilin-1 PS1 and 2
Aβ: amyloid-β
BACE: β-secretase
sAPPβ: Soluble amyloid precursor proteins beta
sAPPα: Soluble amyloid precursor proteins alpha
CTFβ or C99: Carboxy-terminal fragment β
CTFγ or AICD: Carboxy-terminal fragment γ or APP intracellular domain
NMDA: N-methyl-D-aspartate
FDA: Food and Drug Administration
Ach: Acetylcholine
AChE: Acetylcholinesterase
M1: Muscarinic receptor 1
Aβ--AChE: Aβ–acetylcholinesterase macromolecular complex
CMA: Chaperone-mediated autophagy
mTORC1/2: Mammalian target of rapamycin complex 1 or 2
ATGs: Autophagy-related genes
Akt/PKB: Phosphoinositide 3-kinase (PI3K) and protein kinase pathway
LC3I or MAP1LC3B: Microtubule-associated protein light chain 3
p62/SQSTM1: sequestosome 1
AMPK: AMP-activated protein kinase
ULK1: Unc-51 like autophagy activating kinase 1 complex
TSC1/2: Tuberous sclerosis complex subunit 2
AVs: Autophagic vacuoles
3-MA: 3-Methyladenine
ECS: Endocannabinoid system
CB1R and CB2R: cannabinoid receptors 1 and 2
AEA: Anandamide
FAAH: fatty acid amide hydrolase
MAGL: Monoglyceride lipase
PPAR)γ: Peroxisome proliferator-activated receptor
CBD: Cannabidiol
PPAR: Peroxisome proliferator-activated receptor
UFS: University of the Free State
TRPV1: Transient receptor potential vanilloid 1
ER: Endoplasmic reticulum
ERK: Extracellular signal-regulated kinase
BChE: Butyrylcholinesterase.
